# Carboxylative efficacy of *trans* and *cis* MK7 and comparison with other vitamin K isomers

**DOI:** 10.1002/biof.1844

**Published:** 2022-05-18

**Authors:** Ilenia Cirilli, Patrick Orlando, Sonia Silvestri, Fabio Marcheggiani, Phiwayinkosi V. Dludla, Nadine Kaesler, Luca Tiano

**Affiliations:** ^1^ Department of Life and Environmental Sciences Polytechnic University of Marche Ancona Italy; ^2^ Biomedical Research and Innovation Platform South African Medical Research Council Tygerberg South Africa; ^3^ Division of Nephrology and Clinical Immunology University Hospital of the RWTH Aachen Aachen Germany

**Keywords:** carboxylation, *cis/trans* isomers, GGCX, phylloquinone, vitamin MK7

## Abstract

Carboxylative enzymes are involved in many pathways and their regulation plays a crucial role in many of these pathways. In particular, γ‐glutamylcarboxylase (GGCX) converts glutamate residues (Glu) into γ‐carboxyglutamate (Gla) of the vitamin K‐dependent proteins (VKDPs) activating them. VKDPs include at least 17 proteins involved in processes such as blood coagulation, blood vessels calcification, and bone mineralization. VKDPs are activated by the reduced form of vitamin K, naturally occurring as vitamin K1 (phylloquinone) and K2 (menaquinones, MKs). Among these, MK7 is the most efficient in terms of bioavailability and biological effect. Similarly to other *trans* isomers, it is produced by natural fermentation or chemically in both *trans* and *cis*. However, the efficacy of the biological effect of the different isomers and the impact on humans are unknown. Our study assessed carboxylative efficacy of *trans* and *cis* MK7 and compared it with other vitamin K isomers, evaluating both the expression of residues of carboxylated Gla‐protein by western blot analysis and using a cell‐free system to determine the GGCX activity by HPLC. *Trans* MK7H_2_ showed a higher ability to carboxylate the 70 KDa GLA‐protein, previously inhibited in vitro by warfarin treatment. However, *cis* MK7 also induced a carboxylation activity albeit of a small extent. The data were confirmed chromatographically, in which a slight carboxylative activity of *cis* MK7H_2_ was demonstrated, comparable with both K1H_2_ and oxidized *trans* MK7 but less than *trans* MK7H_2_. For the first time, a difference of biological activity between *cis* and *trans* configuration of menaquinone‐7 has been reported.

**Abbreviations**
GGCX: γ‐glutamylcarboxylase
Gla: γ‐carboxyglutamate
Glu: Glutamate
K1H2: Reduced phylloquinone
MK4H2: Reduced menaquinone‐4
MK7H2: Reduced menaquinone‐7
MKs: Menaquinones
nHDFs: neonatal Human Dermal Fibroblasts
TRLs: triglyceride rich lipoproteins
VKDPs: vitamin K‐dependent proteins
VKORC1: vitamin K 2,3‐epoxide reductase complex subunit 1

## 1 INTRODUCTION

Vitamin K represents a group of fat‐soluble compounds sharing a common chemical structure, 2‐methyl‐1,4‐naphthoquinone, and differ in length and in the saturation degree of the side chain.^1^ Besides vitamin K3 (menadione), which is of synthetic origin, vitamin K1 (phylloquinone) and K2 (menaquinone) are the main forms found in nature. Particularly, phylloquinone represents about 75%–90% of all vitamin K consumed in the human diet^2^ and it is found in leafy green vegetables, mainly in kale, collard green, spinach, and broccoli.^3^ Conversely, vitamin K2 is less common and it is also produced by gut bacteria. Menaquinone (MKs) comprises 10 different subtypes in relation to the length of their side chain, ranging from MK4 to MK13. The most relevant menaquinones in the diet are MK4 and MK7. The first is the only form not synthesized by bacteria, but instead is the product of tissue‐specific conversion directly from phylloquinone^4^ although it is also found in some animal products like chicken, egg yolks, and butter. MK7 is particularly abundant in food natto, a popular Japanese dish obtained by soybean fermentation using *Bacillus subtilis natto*.

The main function of all types of vitamin K is to activate proteins that serve important roles in blood clotting, heart, and bone health. In particular, the reduced form of vitamin K is a cofactor for γ‐glutamylcarboxylase (GGCX), an enzyme catalyzing the reaction of post‐translational carboxylation of glutamate (Glu) residues into γ‐carboxyglutamate (Gla) of the vitamin K‐dependent proteins (VKDPs) activating them. All the VKDPs must be carboxylated to become active and the most well‐known include coagulation factors II, VII, IX, X, prothrombin, proteins C, S, Z, and the bone‐related proteins (matrix gla protein, gla‐rich protein, and osteocalcin).^5^ Therefore, VKDPs are involved in processes such as the regulation of the blood coagulation pathway, preventing calcification of blood vessels, and increasing bone mineralization.

As a result of the carboxylation, vitamin K is transformed to an epoxide which is reduced back by the vitamin K 2,3‐epoxide reductase complex subunit 1 (VKORC1).^6^ VKORC1 represents the main target of warfarin, the most used anticoagulant drug, which prevents the reduction of vitamin K, promoting a decrease of all VKDPs carboxylation, including the coagulation factors.

However, vitamin K1 and K2 exhibit different chemical characteristics leading to a difference in absorption and tissue distribution, therefore affecting the biological function. In fact, after the transportation of all vitamin K in the liver by triglyceride‐rich lipoproteins (TRLs), K1 is retained and rapidly excreted while K2 is transported in circulation by LDL to extrahepatic tissues such as bone and vasculature. Therefore, vitamin K2 has a better absorption profile in comparison with K1, although it varies between isoforms.^7^ Among these, MK7 seems to be the most efficient in terms of bioavailability and biological effect.^8^ In fact, comparing the adsorption between MK7 and K1, administrated through natto and spinach, respectively, menaquinone exhibited 10‐fold higher postprandial serum concentration in comparison with phylloquinone, and a half‐life of 72 h with respect to 3 h of phylloquinone, lasting up to 144 h in the circulation.^9^

In addition, the role of MK7 in bone health has been highlighted by several studies showing how natto consumption reduces the incidence of hip fractures in women in Japan^10^, ^11^ and recently confirmed by a large prospective cohort study.^12^ This evidence, along with a large set of clinical studies, highlights the beneficial effects of MK7 supplementation in the prevention of osteoporosis^13^, ^14^, ^15^ and in the reduction of vascular stiffness,^16^, ^17^ making MK7 essential for the prevention of bone and cardiovascular disease (CVD). Therefore, its supplementation is recommended in developmental stages and in those who have a CVD and osteopenic risk.

The production of K vitamers, including MK7, can occur by natural fermentation or by chemical synthesis. Chemical synthesis is a more economical method. The fermentation is carried out by highly specific enzymes and produces all *trans* K vitamers, whereas by chemical synthesis both isomeric forms (*cis* and *trans*) are produced in a variable proportion dependent from the method of synthesis used. Moreover, the industrial production includes a purification phase of the post‐reaction mixture and, depending on the method and on the efficiency, the final preparation may contain both forms.^18^ However, geometric isomerization of isoprenoid units can occur also during incorrect technological processes and storage conditions, mainly by UV radiation, oxygen, and high temperature exposure, leading to radical formation and molecule oxidation, and consequently to the formation of epoxides and *cis* isomers at different locations in the isoprenoid chain of MK7.^18^

The presence of different forms of vitamin K2, including *cis/trans* isomers is very important taking into consideration the biological properties of the vitamin preparations, because the impact on humans is unknown. Moreover, some studies demonstrated that the *cis* isomer of the vitamin K1 is inactive or exhibits merely 1% of the biological activity of the *trans* forms and is present in supplemented food, in particular in infant formulas.^19^, ^20^, ^21^ On the contrary, although the interest and the use of MK7 has grown considerably in the last decade, there is still no specific evidence on the activity of the *cis* isomer compared with the *trans* of this menaquinone. In this context, the present study aims to compare the carboxylative efficacy of both *cis* and *trans* MK7 isomeric forms.

## 2 EXPERIMENTAL PROCEDURES

**FIGURE 1:**
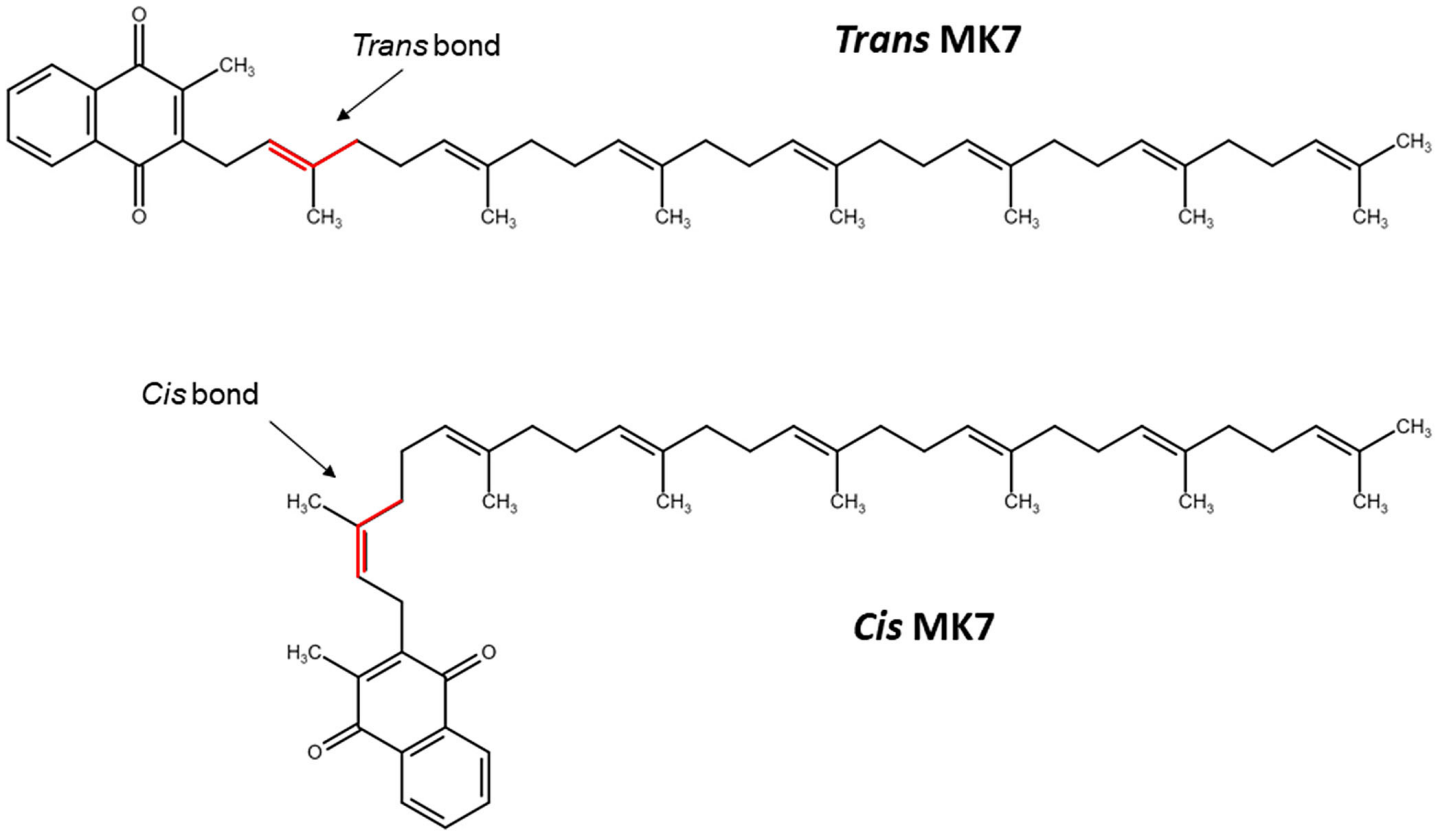
Chemical structure of *trans* and *cis* conformations of menaquinone‐7 (MK7)

The carboxylative efficacy of *cis* and *trans* isomeric forms of MK7 (Figure 1) has been compared to evaluate both the synthesis of residues of carboxylated Gla‐protein by western blot analysis and using a cell‐free system, to determine the vitamin K‐dependent gamma‐glutamyl carboxylase (GGCX) activity by HPLC. *Trans* (≥99%) and *cis* (≥95%) MK7 were kindly provided by Gnosis‐Lesaffre (Milan, Italy), while MK4, K1, and warfarin were purchased from Sigma‐Aldrich.

### 2.1 Cell culture and treatment

Primary culture of neonatal Human Dermal Fibroblasts (nHDFs), purchased from Lonza Group Ltd., were maintained in Minimum Essential Medium (MEM) supplemented with 10% fetal bovine serum (Carlo Erba reagents) penicillin (100 U/ml), streptomycin (100 μg/ml), and L‐glutamine (2 mM), at 37°C under controlled humidified atmosphere containing 5% CO_2_.

For all experiments, confluent cells at the passages 11–14 were treated and incomplete medium was used to avoid the interference of serum K vitamins. After the verification of the warfarin treatment condition efficient on Gla‐protein inhibition (200 μM for 24 h), cells were incubated for 24 h in the presence of warfarin 200 μM and different concentrations of *trans* MK7 (0.1, 1, 10, 50 μM), to find the lowest concentration effective in carboxylation restoring. Subsequently, MK7 isomers were compared by treating nHDFs for 24 h with 1 and 10 μM of *cis* or *trans* MK7 in combination with warfarin (200 μM).

Treated cells were scraped in cold PBS, centrifuged (1000 g for 5 min at 4°C) and lysed in RIPA buffer (140 mM NaCl, 10 mM Tris–HCl pH 8, 0.1% SDS, 0.1% sodium deoxycholate, 1% Triton X‐100, 1 mM EDTA, 0.5 mM EGTA) and added to a protease inhibitor cocktail.

### 2.2 Western blot analysis

Protein concentration was quantified using a BCA protein assay kit (ThermoFisher Scientific) and 35 μg of denaturated proteins were resolved on 10% SDS–PAGE and then transferred onto polyvinylidene fluoride membrane. The membranes were blocked in Tris‐buffered saline with 0.2% (v/v) Tween 20 (TBS‐T) containing 5% (w/v) nonfat dry milk, and then exposed to the primary antibody anti‐γ‐carboxyglutamyl (Gla) residues (1 μg/ml; BioMedica Diagnostics) or anti‐β‐actin (1:8000; Santa Cruz Biotechnology) for 2 h at room temperature. After washing in TBS‐T, the membranes were incubated with the secondary antibody anti‐mouse IgG, HRP‐linked (1:4000; Cell Signaling Technology) for 1 h at room temperature. Protein bands were acquired by C‐DiGit Blot Scanner (LI‐COR Biosciences) using SuperSignal West Dura ECL (ThermoFisher Scientific) and quantified by densitometry using the ImageJ software. β‐Actin was used as a reference control.

### 2.3 GGCX activity

The ability of *cis* and *trans* MK7 forms to promote γ‐glutamyl carboxylase activity was evaluated chromatographically by using an hexapeptide (FLEFLK) conjugated with fluorescein marker (FITC) containing only one carboxylation site as reported by the method previously described.^22^ Vitamin K1, MK4, and *cis* and *trans* MK7 isomers were chemically reduced by incubation in a mixture of 20 mM DTT, 50 mM NaCl, and 2 mM Tris at 37°C for 24 h in the dark and added to a microsomal fraction extracted from the livers of five mice fed for 4 weeks with a low vitamin K diet (K1, 5 μg/Kg) containing GGCX and to a FLEFLK‐FITC fluorescent peptide. HPLC analysis with fluorescence detection optimized for FITC (494 nm excitation and 521 nm emissions) enabled the carboxylated and the undercarboxylated forms to be discriminated. The efficacy of each vitamin K reduction was measured by reversed‐phase HPLC by separation on a C12 Max RP (Phenomenex, Germany) against isocratic methanol and detection at 246 nm, as described earlier.^23^ GGCX activities were calculated in relation to reduced phylloquinone (K1H2) that was originally used in the methodological paper and reduced MK4 was used as a positive control.

### 2.4 Statistical analysis

All experiments were performed at least in triplicate. Data are presented as the mean ± standard deviation. Statistical analyses were conducted using GraphPad Prism version 8.4.2. Comparisons among groups were analyzed using one‐way ANOVA followed by Tukey's multiple comparisons test for GGCX activity and using unpaired T test for GLA‐protein content. A *p* value of ≤0.05 was considered statistically significant and reported as: *p ≤ 0.05; **p ≤ 0.01; ***p ≤ 0.001.

## 3 RESULTS

Efficacy in promoting carboxylation by different forms of vitamin K could be evaluated in a cellular model by blocking the recycling of vitamin K already contained in the cells as this could mask the results. This is possible using vitamin K antagonists, such as warfarin, which inhibit the enzyme vitamin K epoxide reductase.

**FIGURE 2:**
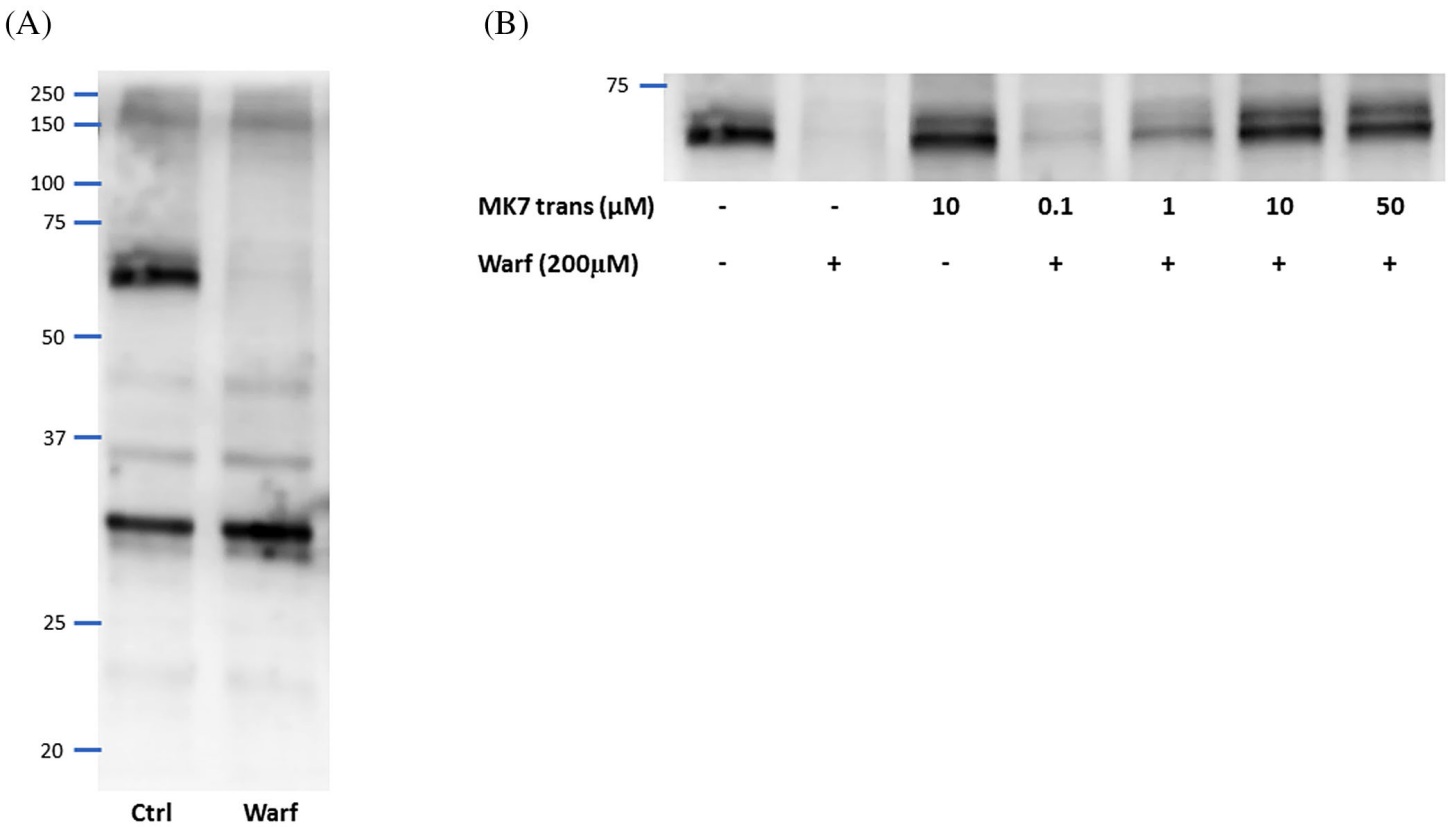
Qualitative western blot of Gla‐protein in nHDF cells. (A) Pattern of Gla‐protein expression after 24 h treatment with 200 μM of warfarin (Warf) compared with untreated cells (Ctrl). (B) 70 kDa Gla‐protein variation after co‐incubation with warfarin (200 μM) and a range of trans MK7 concentration

In particular, 24 h treatment with 200 μM of warfarin of nHDF cells decreased carboxyglutamyl (GLA) residues of a single unknown protein band of approximately 70 kDa (Figure 2). In order to evaluate the concentration of *trans* MK7 able to counteract the warfarin effect, four different concentrations (0.1, 1, 10 and 50 μM) of this vitamin were tested. Figure 2B shows that the lower concentration of *trans* MK7 was not enough to restore the carboxylation of the 70 kDa GLA‐protein inhibited by 200 μM warfarin. It was partially restored by adding 1 μM of exogenous *trans* MK7 and was totally rescued in the presence of 10 μM *trans* MK7, while further GLA‐protein increment did not occur with higher MK7 concentration (Figure 2B).

**FIGURE 3:**
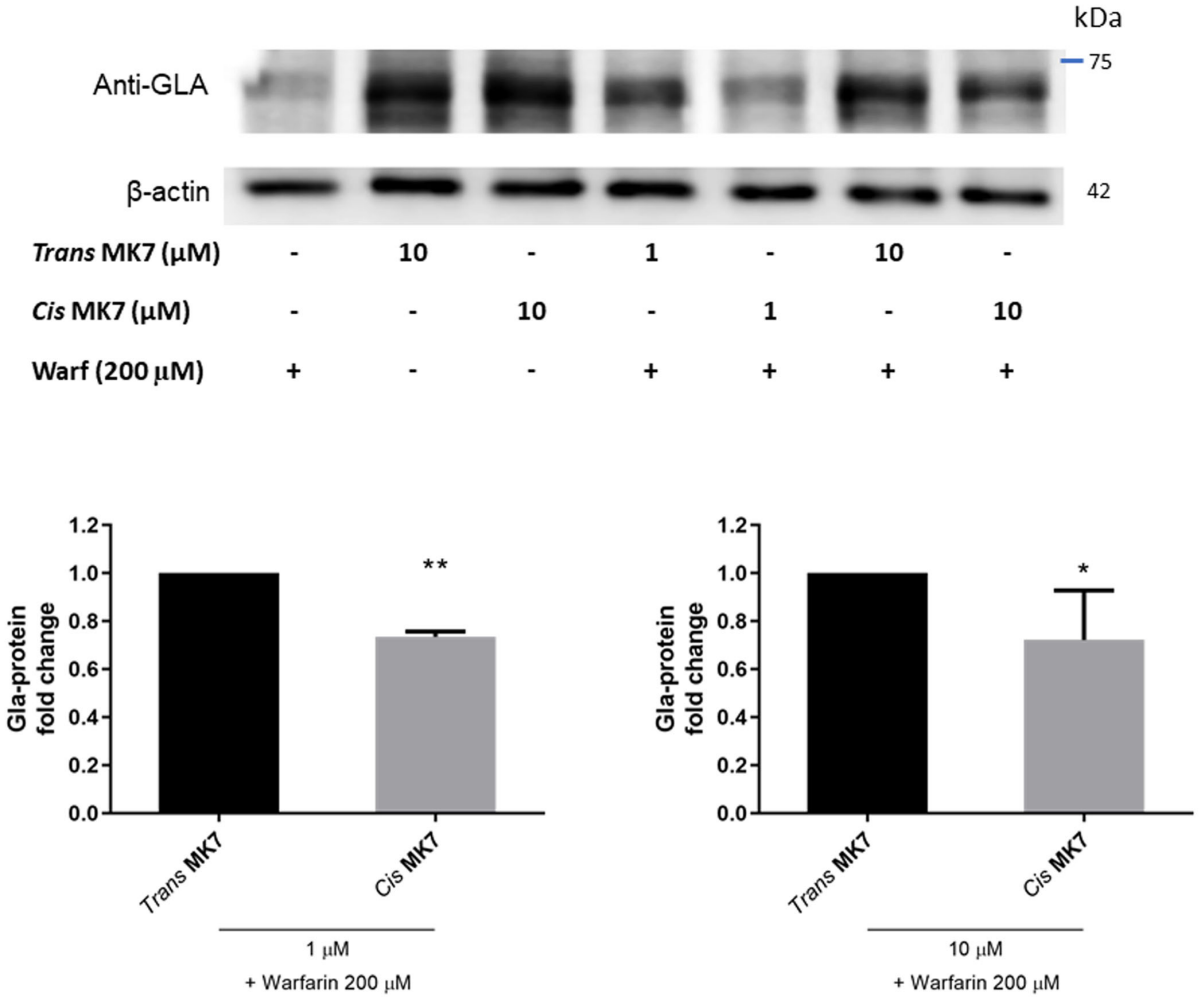
Western blot and densitometric analysis of about 70 kDa Gla‐protein in nHDFs treated for 24 h with warfarin (200 μM) and/or MK7 in *trans* or *cis* conformation (1 and 10 μM). Protein levels are reported as Gla‐protein fold change in cells treated with the same concentration of *cis* vs *trans* MK7. Data were normalized to β‐actin. Data are represented as mean ± *SD*. *T* test: *p ≤ 0.05; **p ≤ 0.01

The carboxylative efficacy of both *cis* and *trans* MK7 isomers were evaluated by co‐incubating nHDF cells with 200 μM of warfarin and MK7 isomers at 1 or 10 μM for 24 h. Lower carboxylation level of the protein at about 70 kDa was observed with the *cis* isomer, compared with *trans* at equal concentrations. In particular, the relative level of 70 kDa Gla‐protein induced by *cis* MK7 was the same for both tested concentrations; 0.73 ± 0.01 and 0.72 ± 0.10 at 1 and 10 μM, respectively (Figure 3).

Carboxylative efficacy was evaluated also by cell‐free system using a synthetic peptide (FLEFLK) conjugated with fluorescein. When its carboxylation site is carboxylated, its retention time changes and can be revealed by HPLC.^22^

Since only the reduced form of vitamin K acts as a cofactor of the carboxylases and is responsible for the activation of the vitamin K‐dependent proteins, to compare the carboxylative activity of different vitamin K isomers, they were chemically reduced before the addition to the microsomal fraction containing GGCX and FLEFLK‐FITC fluorescent peptide. Therefore, the carboxylative efficacy is dependent on the amount of reduced forms of vitamin K isomers used and reported in Table 1.

**TABLE 1:** Percentage of reduced form of K1, MK4, *cis* MK7, and *trans* MK7 used in the GGCX activity assay

Vitamin K isomers	Reduction
K1	90%
MK4	100%
*Cis* MK7	61%
*Trans* MK7	44%

**FIGURE 4:**
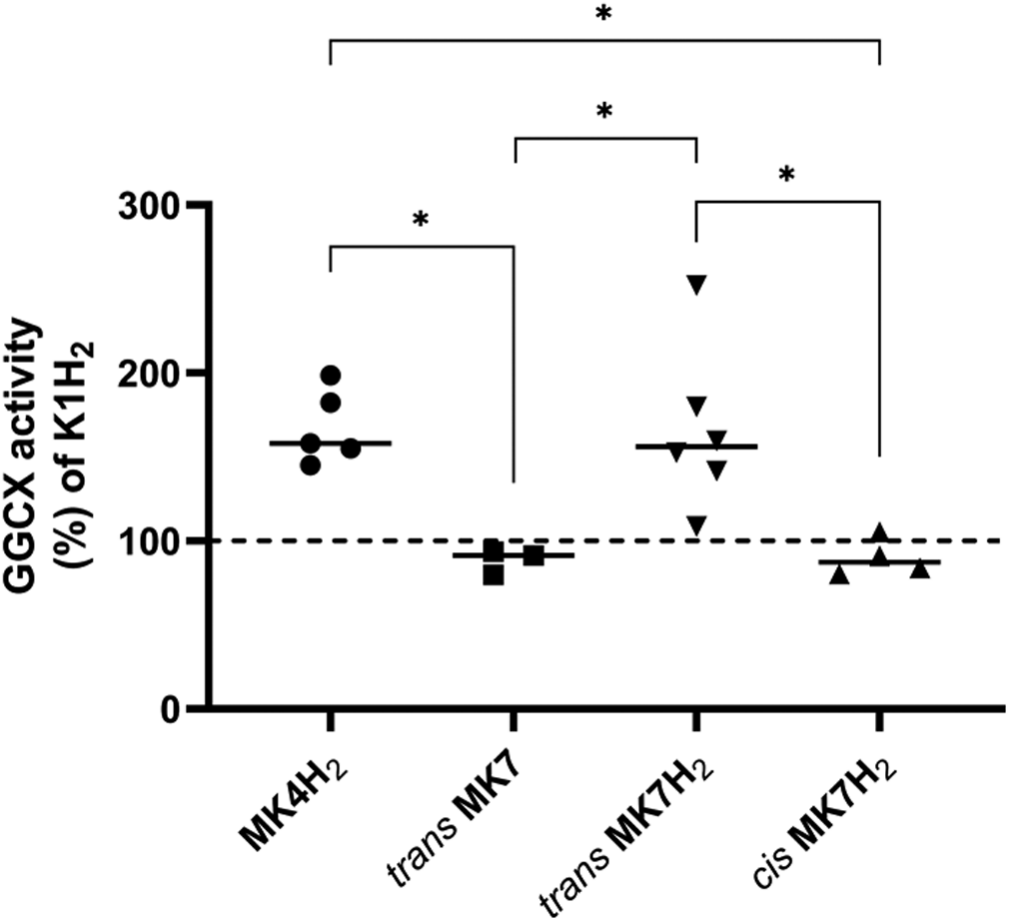
GGCX activity of reduced MK4 (MK4H2), oxidized *trans* MK7 (*trans* MK7), reduced *trans* MK7 (*trans* MK7H2), and reduced *cis* MK7 (*cis* MK7H2) expressed as percentage of reduced vitamin K1 (KH2) activity. *p ≤ 0.05

As shown in Figure 4, MK4H_2_ and MK7H_2_ promoted the highest carboxylative activity reaching values, expressed as a percentage of GGCX activity with respect to K1H_2_, of 168 ± 22% and 166 ± 48%, respectively. However, MK4 along with K1 had the highest stability in their quinol form, being predominantly present in the reduced form (100% and 90%, respectively). Conversely, carboxylative activity of *trans* MK7 is attributable to only 44% of its reduced form. Finally, *cis* MK7H_2_ showed a comparable GGCX activity to both oxidized *trans* MK7 and K1H_2_ (90 ± 11% and 88 ± 7%, respectively), significantly lower compared with *trans* MK7H_2_ (*p* ≤ 0.01).

## 4 DISCUSSION

Vitamin K can exist as geometric isomers, *cis* and *trans*, resulting in a non‐linear and linear molecular structure, respectively. The double bond arrangement determining the molecular shape affects the interaction with subcellular structures such as GGCX and, consequently, the biological activity.^24^

It was demonstrated that only the *trans* configuration of vitamin K1^20^ has biological activity. Nevertheless, Matschiner JT and Bell RG found a similar uptake and turnover of *cis* and *trans* phylloquinone in different tissues of vitamin K deficiency‐induced rats following intracardiac injection of both isomers.^25^ Conversely, another study showed differences in metabolism and distribution. In particular, Knauer et al. showed that the concentration of the *cis* isomer of vitamin K1 in liver decreased much slower in comparison with the *trans* one. Moreover, the *cis* isomer was associated with the mitochondrial fraction, while the *trans* configuration with the endoplasmic reticulum fraction.^19^ However, until now, no biological data are available on the biological activity of the geometric menaquinone isomers.^26^

In this context, the present study aimed to compare the vitamin MK7 isomers efficacy in promoting carboxylation, evaluating both the expression of residues of carboxylated Gla‐protein by western blot analysis and using a cell‐free system to determine the vitamin K‐dependent gamma‐glutamyl carboxylase (GGCX) activity by HPLC.

Firstly, carboxylation deficit was created by treating the fibroblasts with warfarin, which in the cellular model and under the experimental conditions blocked the carboxylation of a single protein band corresponding to about 70 kDa, which was analyzed in relation to different forms of MK7. In this cellular model, *trans* MK7 showed a higher ability to carboxylate the 70 KDa GLA‐protein, previously inhibited by warfarin, compared with *cis* isomer independently from the concentration used. However, *cis* MK7 co‐supplemented with warfarin was found to induce a carboxylative activity albeit of small extension. This could be due to the real capacity of this isomeric form to interact with vitamin K‐dependent gamma‐glutamyl carboxylase. Alternatively, since vitamin K is recycled, the possible presence of residual *trans* form could be enough to activate the carboxylation.^27^ These data are confirmed chromatographically by determining GGCX activity in a rat microsomial fraction, in which a slight carboxylative activity of *cis* MK7H_2_, comparable with both K1H_2_ and oxidized *trans* MK7, has been showed. The latter is inactive as it must be reduced to act as a cofactor, but probably still retains some type of carboxylative activity although to a lower extension.^28^ However, in our experimental conditions the efficiency of reduction of K vitamers was not identical. In particular, while MK4H_2_ and *trans* MK7H_2_ showed a similar and the highest carboxylative capacity, the amount of reduced *trans* MK7 (44%), the active form, was lower compared with MK4H_2_ (100%).

In this regard, Buitenhuis et al.^28^ evaluated the ability of phylloquinone, menadione (K3), and different menaquinones (MK1 to MK10) to serve as a cofactor for the hepatic vitamin K‐dependent carboxylase by determining their kinetic constants. According to our results, the authors showed the higher carboxylative activity of menaquinones compared with K1, while menadione was not active. Conversely, they suggested that at increasing isoprenoid units, the half‐maximal reaction velocity decreased, resulting in a higher enzymatic activity associated with MK4 compared with MK7. The relationship between activity and isoprene chain length was also investigated in in vivo studies showing that serum undercarboxylated osteocalcin level decreased after administration of 100 μg MK7 for 12 weeks^14^ and 600 μg of MK4 for 5 weeks.^29^ Since none of the previous authors discriminated the isomeric form used in their study, the opposite results could be related to a different configuration of menaquinones used. Therefore, the different ratio of *cis/trans* in the composition could have affected the carboxylative activity of the menaquinones.

*Cis* isomers of MK7 are formed during the chemical synthesis of this vitamin.^30^ In addition, geometric isomerization can occur following incorrect technological processes and storage conditions such as light, oxygen, and high temperature exposure which can trigger molecule oxidation and the formation of epoxides and *cis* isomers at different locations in the isoprenoid chain of MK7.^21^, ^24^, ^31^ In this respect, comparative analysis of different formulas has shown that the MK7 impurity content and the level of *cis* isomers could be remarkably high, both leading to increased instability and lower biological effect.^4^, ^18^

Therefore, the supplements can contain both the *cis* and *trans* forms of MK7. Due to the identification of a high content of the *cis/trans* MK7 isomers in reference to all *trans* MK7 in some dietary supplements,^18^ the determination of the biological activity as well as the toxicity of the *cis/trans* forms of MK7 is needed.^32^

Since the vitamin K supplementation is essential to reach the recommended daily intake in certain conditions such as during developmental stages, CVD, and osteopenia, and, considering that the *cis* isomers have lower biological effects, the isomeric composition must be taken into consideration for the supplement formulation and its efficacy.

In conclusion, for the first time a difference of biological activity between *cis* and *trans* configuration of menaquinone‐7 has been reported. In particular, the *trans* isomeric form showed higher carboxylative capacity compared with the *cis* one. However, this latter was not completely ineffective. This could be as a consequence of a conserved capacity of interaction with enzymes even if to a lower extension and/or of the presence of trans residual contaminant traces.

## CONFLICT OF INTEREST

No conflict of interest to declare.

## Data Availability

The data that support the findings of this study are available from the corresponding author upon reasonable request.
